# Stimulation priming and psychological state shape functional connectivity following prefrontal theta-burst stimulation

**DOI:** 10.1162/IMAG.a.1267

**Published:** 2026-06-04

**Authors:** Stefanie De Smet, Guo-Rong Wu, Debby C.W. Klooster, Beatriz Catoira, Sara De Witte, Lais B. Razza, Vincent van de Ven, Alexander T. Sack, Chris Baeken, Marie-Anne Vanderhasselt

**Affiliations:** Ghent Experimental Psychiatry (GHEP) lab, Faculty of Medicine and Health Sciences, Department of Head and Skin, Ghent University, Ghent, Belgium; Brain Stimulation and Cognition (BSC) Lab, Department of Cognitive Neuroscience, Faculty of Psychology & Neuroscience, Maastricht University, Maastricht, The Netherlands; Department of Cognitive Neuroscience, Faculty of Psychology and Neuroscience, Maastricht University, Maastricht, The Netherlands; Key Laboratory of Cognition and Personality, Faculty of Psychology, Southwest University, Chongqing, China; Care & Cure Lab of the Electromagnetics Group (EM4Care+Cure), Department of Electrical Engineering, Eindhoven University of Technology, Eindhoven The Netherlands; Department of Psychiatry, Vrije Universiteit Brussel (VUB), Universitair Ziekenhuis Brussel (UZ Brussel), Brussels, Belgium; Department of Psychiatry and Neuropsychology, School for Mental Health and Neuroscience (MHeNs), Brain+Nerve Centre, Maastricht University Medical Centre+ (MUMC+), The Netherlands

**Keywords:** theta burst stimulation (TBS), stimulation priming, dorsolateral prefrontal cortex (DLPFC), resting-state connectivity, functional magnetic resonance imaging (fMRI)

## Abstract

Intermittent theta-burst stimulation (iTBS) is increasingly used to neuromodulate prefrontal brain regions, such as the dorsolateral prefrontal cortex (DLPFC), allowing to effectively change local excitability in research settings and to optimize therapeutic outcomes. However, response variability remains high, and its clinical efficacy is modest. Priming with theta-burst stimulation (TBS), in which a preconditioning TBS protocol precedes a test iTBS protocol, has been proposed to enhance stimulation efficacy and reduce inter-individual variability, particularly when combining protocols that are expected to induce different changes in cortical excitability. Yet, its underlying neurobiological effects remain unclear. In this within-subjects study, we investigated the effects of TBS priming on resting-state functional connectivity in 47 healthy subjects. Each participant completed four counterbalanced sessions, 1 week apart, receiving either a control condition with sham priming and sham test stimulation, an iTBS-alone condition (without priming), a priming condition where iTBS preceded iTBS, or a priming condition where continuous TBS (cTBS) preceded iTBS. Resting-state scans were acquired before and after each stimulation condition, alongside assessments of self-reported mood and perseverative thinking. Contrary to our expectations, priming prefrontal iTBS with cTBS resulted in reduced connectivity changes in both regions near the stimulation site and more distal temporal areas, and priming with iTBS significantly reduced connectivity changes in prefrontal regions, both relative to iTBS alone. Changes in perseverative thinking further moderated stimulation effects, with greater increases associated with stronger decreases in left prefrontal connectivity following cTBS-iTBS priming. These findings highlight the complex effects of prefrontal TBS priming and its interaction with psychological states, underscoring the importance of accounting for state dependency in neuromodulation research.

## 1 Introduction

Over the past decades, repetitive transcranial magnetic stimulation (rTMS) has gained increasing attention in both research and clinical practice as a non-invasive brain stimulation (NIBS) technique for modulating cortical excitability (Baeken et al., 2019). By delivering brief magnetic pulses to targeted brain regions, rTMS can induce neuroplastic changes that persist beyond the stimulation period. A specific form of rTMS, theta burst stimulation (TBS), delivers patterned bursts of three pulses at 50 Hz, repeated every 200 milliseconds, which has been shown to induce plasticity in neural circuits (Huang et al., 2005). Evidence from the motor cortex suggests that intermittent TBS (iTBS) is associated with excitatory, long-term potentiation (LTP) like effects, while continuous TBS (cTBS) is linked to temporarily suppressive neuronal activity through long-term depression (LTD) mechanisms (Huang & Rothwell, 2007; Suppa et al., 2016), though these effects can vary depending on stimulation parameters and individual factors (e.g., De Smet, Int-Veen, et al., 2024; McCalley et al., 2021; also see Hussain & Freedberg, 2025). When applied to the dorsolateral prefrontal cortex (DLPFC), TBS is a promising approach for the treatment of depression (Trapp et al., 2025; Voigt et al., 2021), offering a shorter and more cost-effective alternative to conventional rTMS protocols (Blumberger et al., 2018; Mendlowitz et al., 2019)). However, despite its therapeutic potential, TBS outcomes remain highly variable, both in clinical settings and in experimental research, with limited (clinical) efficacy across individuals (Chu et al., 2020; Corp et al., 2020; Schilberg et al., 2017). This variability highlights the need for exploring strategies to enhance the effectiveness and reliability of TBS protocols.

A key factor contributing to the variability in TBS outcomes is the brain’s functional state at the time of stimulation, also referred to as brain-state dependency. A growing body of research demonstrates that rTMS effects interact with ongoing brain states (reflecting underlying neural activity as well as psychological states), and that uncontrolled variability in these states, both across individuals and across sessions, can lead to inconsistent outcomes (Bergmann, 2018; Sack et al., 2024; Schutter et al., 2023). One proposed strategy to address this is to precondition the brain state prior to TBS using a priming protocol. Stimulation priming involves applying a prime NIBS protocol before the main (test) protocol, with the aim of enhancing its effects. This approach is based on the concept of metaplasticity, which refers to the modulation of synaptic plasticity based on prior neuronal activity (Müller-Dahlhaus & Ziemann, 2015).

Evidence for such priming effects is well established in the motor cortex. Studies show that priming-test configurations, particularly those combining protocols associated with LTD- and LTP-like plasticity, elicit stronger and potentially more reliable neuroplastic changes than test protocols alone (Hassanzahraee et al., 2018). Moreover, meta-analytical evidence indicates that using cTBS as a primer before iTBS amplifies iTBS-induced increases in motor cortex excitability (Hassanzahraee et al., 2018). This effect aligns with homeostatic metaplasticity, where an inhibitory prime reduces prior postsynaptic activity, shifting the LTD/LTP threshold to favor LTP-like plasticity and strengthening the impact of iTBS. Similarly, corticospinal reactivity can be modulated by priming with TBS protocols commonly associated with facilitatory effects, such as iTBS (Mastroeni et al., 2013). Yet, evidence suggests that priming with the same type of stimulation, such as iTBS followed by iTBS, can lead to a weakening or even reversal of the expected after-effects (Mastroeni et al., 2013; see also Sack et al., 2024). This indicates that priming protocols may be most effective when they involve stimulation protocols expected to induce different excitability changes.

Importantly, emerging evidence suggests that similar priming principles may also apply beyond the motor system, including the prefrontal cortex. For instance, Fitzgerald et al. (2008) demonstrated that a short high-frequency prime preceding a low-frequency rTMS protocol over the prefrontal cortex led to a greater reduction in depressive symptoms in patients with treatment-resistant depression compared to the test protocol alone. Complementing these findings, a network meta-analysis evaluating different NIBS techniques for the acute treatment of depression found that rTMS priming protocols (where low-frequency rTMS is preceded by a brief period of low-intensity high-frequency stimulation) have the second highest probability of being efficacious, with only bitemporal electroconvulsive therapy showing superior response efficacy (Mutz et al., 2019). As in the motor cortex, the effectiveness of these prefrontal priming approaches appears to rely on combining stimulation protocols typically associated with different plasticity profiles (i.e., LTP vs LTD-like). Given the increasing clinical adoption of TBS protocols as time-efficient alternatives to conventional rTMS, and building on the metaplastic mechanisms established in the motor cortex, this provides a conceptual rationale for examining how different TBS protocols (i.e., cTBS and iTBS) interact when applied sequentially over the prefrontal cortex. However, given that therapeutic protocols often consist of repeated iTBS sessions (De Witte et al., 2025), it remains clinically and theoretically valuable to investigate priming approaches involving the repetition of the same stimulation protocol. Moreover, comparing the effects of priming protocols using the same or different prime-test configurations may offer important insights into which approaches most effectively enhance prefrontal stimulation outcomes and reduce variability. Overall, these findings suggest that TBS priming (i.e., applying a prime TBS protocol to enhance the effects of a test TBS protocol) may be a promising strategy to optimize iTBS targeting the DLPFC. Investigating its mechanisms of action, and comparing different types of prime-test configurations, can be valuable to further improve treatment protocols.

Neuroimaging methods like functional magnetic resonance imaging (fMRI) provide a powerful tool to explore the neurobiological effects of TBS, offering insights into its mechanisms of action and potential avenues for refining stimulation strategies (Bergmann et al., 2016). In recent years, resting-state functional connectivity has emerged as a valuable approach for assessing functional brain changes following stimulation, particularly in the context of depression (Fox, Halko, et al., 2012; Tura & Goya-Maldonado, 2023). Previous work has examined baseline connectivity biomarkers associated with successful therapeutic responses to rTMS (Klooster et al., 2024), and explored connectivity changes following iTBS over the left DLPFC in healthy individuals (Kirkovski et al., 2023). These studies suggest that iTBS can modulate functional connectivity between the DLPFC and regions implicated in affective processing, including the anterior cingulate cortex (ACC, (Iwabuchi et al., 2017; Singh et al., 2020; Tang et al., 2019). More recent interleaved iTBS-fMRI work has further demonstrated engagement of prefrontal regions during iTBS, while also highlighting substantial variability across sessions and populations (Chang et al., 2024). Overall, these findings indicate that iTBS-induced changes in resting-state functional connectivity are dynamic and shaped by various influencing factors (see also Kirkovski et al., 2023). To date, most neuroimaging studies have focused on the effects of iTBS applied in isolation, leaving the impact of prefrontal TBS priming protocols on resting-state functional connectivity largely unexplored. By engaging metaplasticity-related mechanisms, priming protocols may offer a more suitable framework for explaining inter-individual variability in TBS outcomes than single-session stimulation. Accordingly, examining how different prefrontal TBS priming configurations modulate iTBS-induced connectivity changes may provide important insights into optimizing stimulation strategies.

In the current study, we evaluated the effects of priming iTBS on resting-state functional connectivity in healthy subjects, using priming protocols that either repeated the same stimulation type (i.e., iTBS-iTBS) or combined different stimulation types (i.e., cTBS-iTBS) in the prime-test configuration. Using a within-subjects design, participants completed four sessions, each 1 week apart, in which they received one of four stimulation conditions (in a counterbalanced order): a control condition with sham priming and sham test stimulation, an iTBS-alone condition, an iTBS-iTBS priming condition, and a cTBS-iTBS priming condition. Resting-state scans were acquired before and after each stimulation condition, and self-reported mood and perseverative thinking were assessed throughout each session. Our primary objective was to identify significant connectivity changes associated with priming conditions. Based on previous research suggesting that priming can enhance neuroplasticity, we hypothesized that priming protocols would result in stronger connectivity changes (compared to iTBS alone), specifically reflecting increases in connectivity, both in regions proximal to the stimulation site and in more distant, functionally connected brain areas, with the largest effects expected following the prime-test configuration combining different stimulation types (i.e., cTBS-iTBS). In addition, we explored whether variability in self-reported mood and perseverative thinking, as indices of psychological state, was associated with stimulation-induced connectivity changes.

## 2 Materials and Methods

The study was approved by the medical ethics committee of Ghent University Hospital (B6702020001141) and conducted in compliance with the latest revision of the Declaration of Helsinki. This research was part of a broader project investigating the effects of stimulation priming on various brain functions. The present manuscript focuses on resting-state connectivity, while other findings will be reported elsewhere (e.g., De Smet, Razza, et al., 2024).

### 2.1 Study sample

Fifty healthy individuals were recruited from the general population to participate in the study. Eligibility was assessed through an online screening. Participants met the following inclusion criteria: (1) aged between 18 and 35 years, (2) right-handed, (3) no history of or current psychiatric or neurological conditions, (4) no cardiovascular diseases, (5) no use of substances or medications influencing mood, cognition, or cardiovascular function (such as antidepressants, benzodiazepines, or Z-drugs), (6) smoking fewer than 10 cigarettes per day, (7) normal or corrected-to-normal vision, (8) no history of severe head trauma, brain surgery, or epileptic seizures, (9) no metal implants or fragments in the head, (10) no cochlear implants or pacemakers, (11) not pregnant, (12) no contraindications for MRI or TMS, and (13) no prior exposure to TMS. Three participants were excluded from the analyses due to excessive motion, resulting in a final sample of 47 participants (68% female, mean age = 22, *SD* = 2.31). See Supplementary Materials for details on participant exclusion.

### 2.2 Procedure

**Fig. 1.**
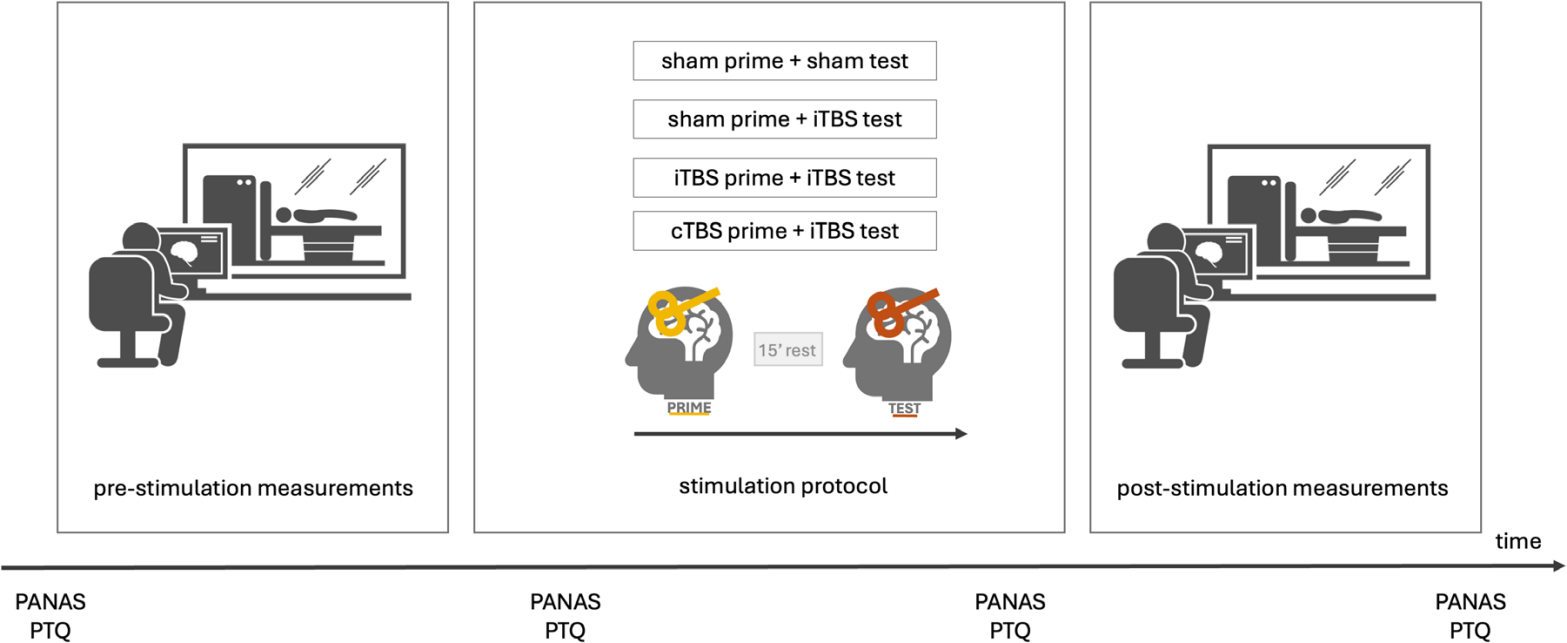
Overview of the experimental procedure. Each session began with a pre-stimulation resting-state scan, followed by one of four prime-test stimulation protocols administered outside the scanner: (1) sham TBS prime followed by sham TBS test (control condition), (2) sham TBS prime followed by iTBS test (iTBS alone), (3) iTBS prime followed by iTBS test (iTBS-iTBS), and (4) cTBS prime followed by iTBS test (cTBS-iTBS). There was a 15-minute interval between prime and test protocol. After stimulation, participants returned to the scanner for a post-stimulation resting-state scan. There was approximately 10 minutes between the end of stimulation and the post-stimulation resting-state scan. Self-reported mood and perseverative thinking were assessed throughout each session. Abbreviations: PANAS, Positive Affect Negative Affect Schedule; PTQ, Perseverative Thinking Questionnaire.

After completing an online eligibility screening, participants were invited to attend four experimental sessions at the Ghent University Hospital, scheduled 1 week apart at the same time of day. They were instructed to maintain sufficient sleep and to avoid strenuous physical activity and alcohol consumption the day before each visit. Additionally, participants were asked to abstain from intense exercise, caffeine, and nicotine for at least 2 hours before each session. During the first session, participants provided written informed consent, and their resting motor threshold was measured to determine the appropriate stimulation intensity. Before the stimulation, baseline assessments were conducted (see Fig. 1), including structural and resting-state scans. Participants were then randomly assigned to one of four priming protocols (see section 2.3), which were administered outside the scanner. Following stimulation, they returned to the scanner for post-stimulation resting-state scans. State measures of mood (i.e., positive and negative affect schedule, PANAS, Watson et al., 1988) and perseverative thinking (i.e., the Perseverative Thinking Questionnaire, PTQ, Ehring et al., 2011) were assessed upon arrival at the lab, before and after the stimulation protocol and at the end of the lab session. At the end of each session, potential adverse effects of the stimulation protocol were evaluated, and participants were asked to guess the stimulation condition they were in. Upon study completion, participants received a compensation of €150.

### 2.3 Neurostimulation

Theta burst stimulation (TBS) was administered using a figure-eight coil (Magstim 70 mm double air film coil) connected to a Magstim Rapid² Plus¹ magnetic stimulator (Magstim Company Limited, Minnesota, USA). Stimulation targeted the left dorsolateral prefrontal cortex (DLPFC) at MNI coordinates x = −38, y = 44, z = 26 (Fox, Buckner, et al., 2012). To ensure accurate targeting, individual anatomical MRI data were used to transform these MNI coordinates into the participant’s native space via an inverse normalization matrix. The TMS coil was positioned over the stimulation site with the aid of the Brainsight neuronavigation system (Brainsight™, Rogue Resolutions, Inc) and was oriented at a 45° angle relative to the brain’s midline.

Each stimulation protocol followed a prime-test configuration, where a priming TBS protocol preceded a test TBS protocol. The test protocol was always administered after the priming protocol. Three priming conditions were used: sham, iTBS, and cTBS, while the test protocols were either sham or iTBS. iTBS was chosen as the test protocol because it is a widely used setup in both research and clinical settings for stimulation over the left DLPFC (e.g., De Smet et al., 2021, De Smet, Int-Veen, et al., 2024; De Witte et al., 2025). This resulted in four stimulation conditions: (1) sham TBS prime followed by sham TBS test (control condition), (2) sham TBS prime followed by an iTBS test (iTBS alone), (3) iTBS prime followed by an iTBS test (iTBS-iTBS), and (4) cTBS prime followed by an iTBS test (cTBS-iTBS; see also Fig. 1). There was a 15-minute interval between the prime and test protocols (De Witte et al., 2025; Duprat et al., 2016; Wittkopf et al., 2021). The iTBS protocol consisted of triplet bursts (three pulses at 50 Hz) delivered in 2-second trains, repeated every 10 seconds for a total of 192 seconds. In contrast, cTBS was administered as a continuous 40-second train of uninterrupted TBS. Stimulation intensity was determined based on each participant’s resting motor threshold (rMT), defined as the minimum TMS intensity required to elicit a motor response in the right abductor pollicis brevis in at least 5 out of 10 consecutive trials (Rothwell et al., 1999). Both iTBS and cTBS were applied at 80% of the individual rMT, with each protocol consisting of 600 pulses (Huang et al., 2005). For sham TBS, the same stimulation parameters as for iTBS were applied (prime and test), but delivered with a placebo version of the Magstim coil. This sham coil mimics the acoustic and somatosensory experience of real stimulation without inducing neurophysiological effects. Participants were naïve to the stimulation procedure and were instructed to keep their eyes closed and wear earplugs throughout the session (De Smet et al., 2021; Duprat et al., 2016). Given that the success rate of correctly guessing the stimulation condition (49.50%) was not significantly different from chance level, *p* = 0.944, the blinding of the stimulation conditions was considered successful. Potential side effects were systematically evaluated following the guidelines outlined by Rossi et al. (2011, 2020). Participants rated their experiences on a 5-point Likert scale across nine possible adverse effects (see also De Smet, Int-Veen, et al., 2024). For a detailed overview of the mean responses to each of the self-report items, as well as comparative statistics between stimulation conditions, see Table S1 in the Supplementary Materials.

### 2.4 Self-report measures

#### 2.4.1 Mood

Participants repeatedly rated their current mood using the Positive and Negative Affect Schedule (PANAS; Watson et al., 1988), which assesses positive and negative affect through adjective-based items. Responses were given on a 5-point Likert scale ranging from “1 = very slightly” to “5 = extremely.” Total scores for each subscale were obtained by summing the respective items, with higher scores indicating greater levels of positive or negative affect.

#### 2.4.2 Perseverative thinking

State changes in repetitive negative thinking, a form of perseverative cognition, was assessed using the Dutch version of the Perseverative Thinking Questionnaire (PTQ-NL; Ehring et al., 2011, 2012). The PTQ is a content-independent measure consisting of 15 self-report items that evaluate participants’ repetitive thoughts about negative experiences or problems. A total PTQ score was calculated by summing all individual items, with higher scores reflecting greater levels of perseverative thinking (see also De Smet et al., 2023).

### 2.5 Image acquisition

Images were collected with a Siemens 3T Magnetom Prisma Fit (Siemens Medical Systems, Erlangen, Germany), using a 64-channel head coil, at the Ghent Institute for Functional and Metabolic Imaging (GIfMI) at the Ghent University Hospital (UZGent). Anatomical imaging consisted of a three-dimensional T1-weighted structural scan (MP-RAGE) with the following parameters: TR = 2250 ms, TE = 4.18 ms, flip angle = 9°, 176 slices, interleaved slice acquisition, field of view = 256 mm, voxel size: 1 x 1 x 1mm, acquisition time = 5:14 minutes. The resting state functional imaging entailed a single echo planar imaging (EPI) sequence with TR = 1720 ms, TE = 27 ms, flip angle = 66°, 52 slices, interleaved slice order, 300 volumes, field of view = 210 mm, voxel size = 2.5 x 2.5 x 2.5 mm, fat saturation, acquisition time = 8:44 minutes. Parallel imaging was performed using GRAPPA with an acceleration factor of 2 and 24 reference lines for autocalibration. During the resting-state scan, participants were instructed to remain still and awake, with their eyes closed. To measure magnetic field inhomogeneities, gradient echo field maps were acquired with two echo times (TE1 = 4.92 ms, TE2 = 7.38 ms) and the following parameters: TR = 520 ms, flip angle = 60°, 52 slices (interleaved order), field of view = 204 mm, voxel size = 2.5 × 2.5 × 2.5 mm, acquisition time = 1:26 minutes.

### 2.6 Data plan

#### 2.6.1 Behavioral analyses

Self-reported changes in mood and perseverative thinking were evaluated using R (version 4.4.0, 2024-04-24, “Puppy Cup”) within the RStudio environment (version 2024.12.0 + 467). Analyses were performed within the linear mixed effects framework using the ‘lmerTest’ (Kuznetsova et al., 2017) and ‘lme4’ (Bates et al., 2015) packages. For all state measures, a linear model was fitted with a random intercept for participants and ‘time’ and ‘stimulation condition’ as the independent variables. Model contrasts were set using sum (i.e., effect) coding schemes. *p*-values were calculated using type-III Wald chi-squared statistics. Multiple comparisons were corrected using the false discovery rate (FDR) method. Effect sizes were reported using eta squared (*η_p_*^2^) and phi (φ) for *F* and *X^2^* test statistics.

#### 2.6.2 Imaging data analyses

Analyses of fMRI data were performed using CONN (Whitfield-Gabrieli & Nieto-Castanon, 2012) release 22.a (Nieto-Castanon & Whitfield-Gabrieli, 2022) and SPM (Penny et al., 2011) release 12.7771. Functional and anatomical data were preprocessed using a modular preprocessing pipeline (Nieto-Castanon, 2020) including creation of voxel-displacement maps, realignment with susceptibility distortion correction using fieldmaps, slice timing correction, outlier detection, indirect segmentation and MNI-space normalization, and smoothing. More detailed information on the preprocessing steps is provided in the Supplementary Materials. Seed-based connectivity maps (SBC) were estimated to characterize whole-brain functional connectivity patterns with the seed area. Functional connectivity strength was represented by Fisher-transformed bivariate correlation coefficients derived from a weighted general linear model (Nieto-Castanon, 2020), modeling the association between the BOLD time series of the seed and each voxel. The seed region was defined as a 10 mm radius sphere centered on the left DLPFC (MNI coordinates: x = −38, y = 44, z = 26) and masked for gray matter using each participant’s anatomical image. To assess differences in stimulation-induced changes, delta maps (post-stimulation minus pre-stimulation SBC maps) were analyzed using a within-subjects one-way ANOVA, implemented in SPM12 via a flexible factorial design. In this model, ‘subject’ was specified as a factor (accounting for repeated measures) and ‘stimulation condition’ as the within-subjects factor (with 4 levels reflecting the different stimulation conditions). The design matrix included a subject-specific intercept to control for interindividual variability. Correction for non-sphericity was applied using restricted maximum likelihood (ReML) estimation to account for potential dependencies between repeated measures. Statistical inference was performed at the whole-brain level, using an *F*-contrast to identify significant main effects of stimulation condition. Results were considered statistically significant using a cluster-wise familywise error (FWE) correction at *p* < 0.05, with uncorrected voxel-wise *p*-values < 0.005. The resulting *F*-map was used as a mask to perform further post hoc analyses using one-sample *t*-tests. To follow up on the significant *F*-test results, we compared iTBS alone to the control condition (i.e., sham prime and sham test protocol). We then tested our hypothesis that priming modulates the effects of iTBS by directly comparing each priming condition (iTBS-iTBS and cTBS-iTBS) to the iTBS-alone condition. Lastly, we directly compared the two priming conditions (cTBS-iTBS versus iTBS-iTBS). The significant statistical parametric map was restricted within the mask with *p* < 0.00625 (i.e., 0.05/8). Anatomical regions were identified using the AAL3 toolbox (Rolls et al., 2020) and labeled based on local maxima.

#### 2.6.3 Exploratory brain-behavior analyses

To examine whether individual differences in self-reported changes in mood and perseverative thinking (i.e., post-stimulation minus baseline) explain variability in stimulation-induced connectivity changes, additional analyses were performed on clusters showing significant effects in the whole-brain analysis. Mean connectivity change values were extracted for each significant cluster. Linear mixed-effects models were fitted with connectivity change as the outcome measure and changes in mood (positive and negative affect) or perseverative thinking, stimulation condition, and their interaction as fixed effects, with a random intercept for participants.

## 3 Results

### 3.1 Self-report measures

The linear mixed-effects analyses revealed no significant interaction effects between time and stimulation condition for any of the self-report measures: negative affect: *X* ^2^(9, *N* = 50) = 11.91, *p* = 0.219, *φ* = 0.49; positive affect: *X* ^2^(9, *N* = 50) = 9.85, *p* = 0.363, *φ* = 0.44; perseverative thinking: *X* ^2^(9, *N* = 50) = 6.45, *p* = 0.694, *φ* = 0.36 (see Fig. S1 in the Supplementary Materials). However, significant main effects of time were observed for all measures, all *X* ^2^’s > 8.56, *p*’s < 0.036, *φ*’s > 0.42 (see Fig. S1 of the Supplementary Materials).

### 3.2 Functional connectivity

**Fig. 2.**
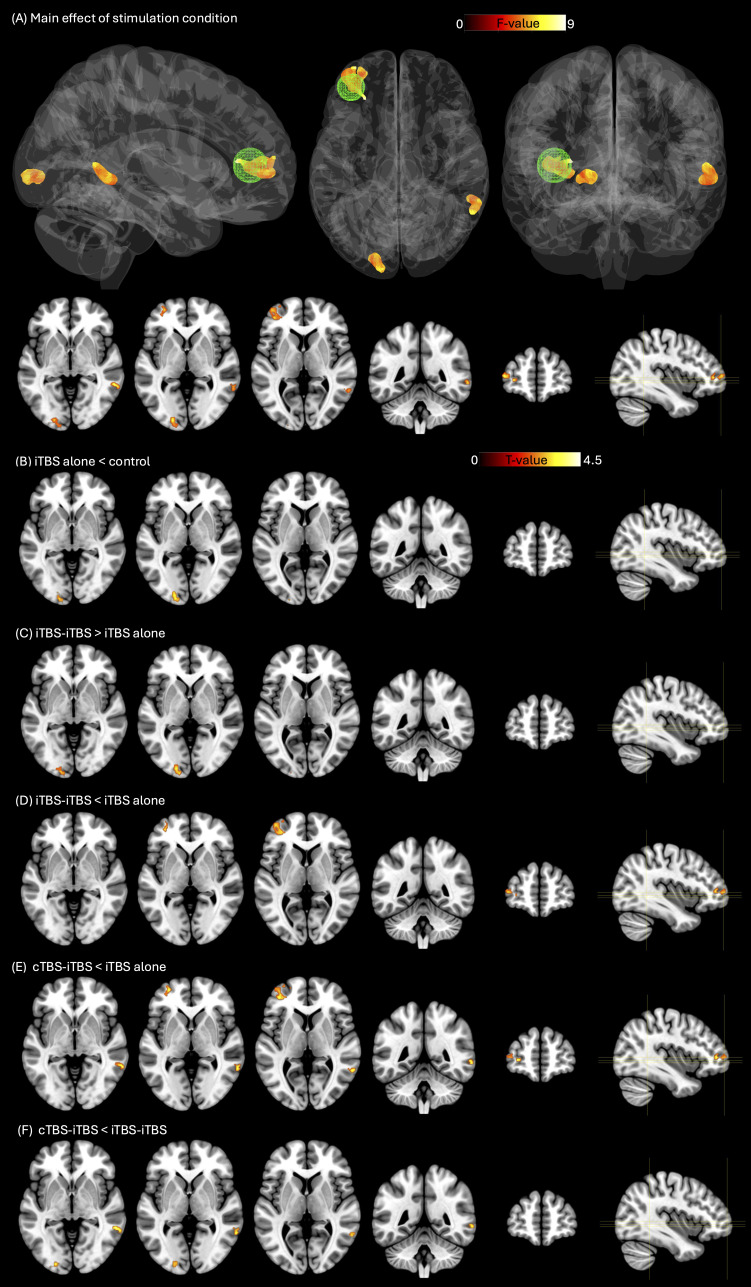
Statistical maps of whole-brain left DLPFC resting-state connectivity. (A) Whole-brain *F*-test results for left DLPFC resting-state functional connectivity, displayed in sagittal, axial, and coronal views. The seed region is marked with a green sphere. The *F*-map was thresholded using cluster-wise familywise error (FWE) correction at *p* < 0.05, with a voxel-wise uncorrected threshold of *p* < 0.005. This *F*-map served as a mask for subsequent post hoc contrasts, which were assessed using *t*-tests with a significance threshold of *p* < 0.006 (0.05/8), including (B) iTBS alone < control, (C) iTBS-iTBS > iTBS alone, (D) iTBS-iTBS < iTBS alone, (E) cTBS-iTBS < iTBS alone and (F) cTBS-iTBS < iTBS-iTBS. See Tables 1 and 2 for full details. Abbreviations: cTBS, continuous theta burst stimulation; iTBS, intermittent theta burst stimulation.

**Fig. 3.**
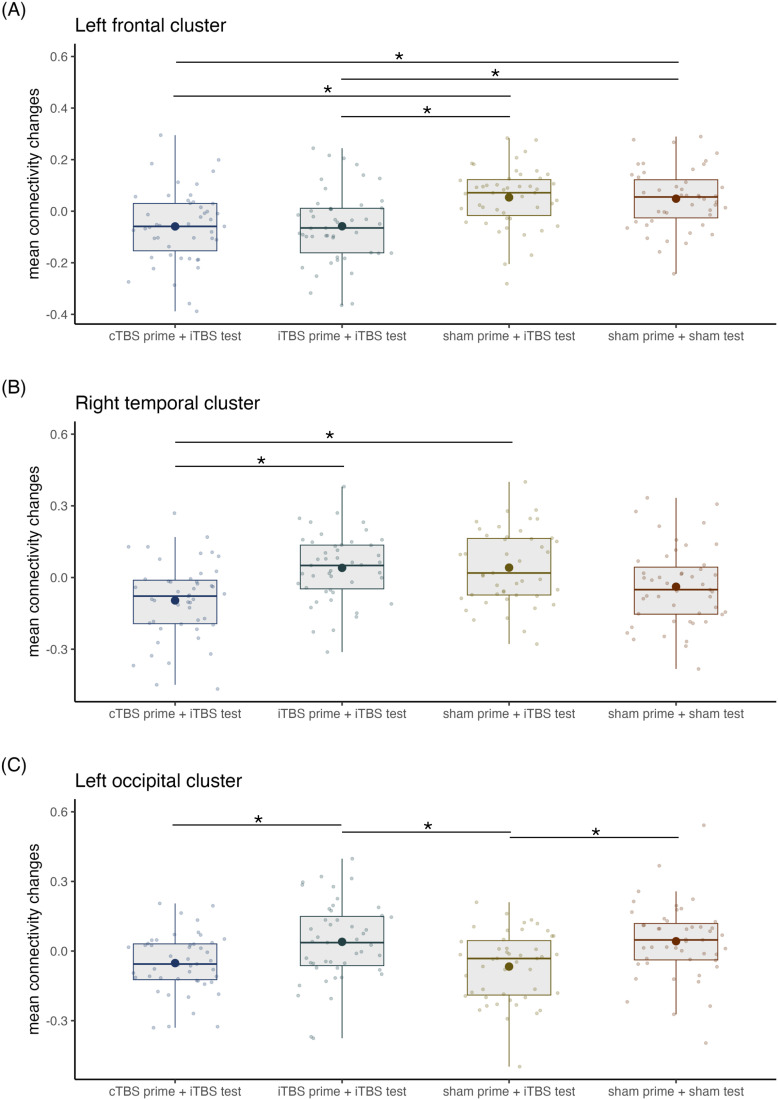
Mean connectivity changes across stimulation conditions for the three significant clusters. Boxplots of mean connectivity changes across stimulation conditions for the significant (A) left prefrontal, (B) right temporal, and (C) left occipital cluster resulting from the *F*-test. Individual data is represented by dots. Each boxplot displays the stimulation condition median alongside the interquartile ranges (horizontal lines). Bold dots in the boxplots represent the mean. Significant differences between stimulation conditions are marked with an asterisk. Abbreviations:: cTBS, continuous theta burst stimulation; iTBS, intermittent theta burst stimulation.

A one-way within-subjects ANOVA revealed significant effects in three different clusters including a left frontal cluster, a right middle temporal cluster, and a left occipital cluster, *F*(3,138) = 4.47, *p* = 0.005, *η*²_p_ = 0.09 (see Table 1 and Fig. 2A). Post hoc comparisons were conducted within these clusters and revealed significant differences in changes in connectivity between stimulation conditions (see Table 2). Whereas there were no significant increases in connectivity changes following iTBS alone compared to the control condition, a significant decrease was observed in the left middle occipital gyrus (Fig. 2B and Fig. 3C). In the same occipital cluster, priming iTBS with iTBS resulted in significantly greater connectivity changes compared to iTBS alone (see Fig. 2C and Fig. 3C). In the left frontal cluster, priming iTBS with both iTBS and cTBS led to significantly lower connectivity changes than iTBS alone (Fig. 2D, 2E and Fig. 3A). This cluster included the left superior frontal gyrus (dorsolateral), middle frontal gyrus and inferior frontal gyrus (triangular part). Similarly, in the right temporal cluster, priming iTBS with cTBS led to significantly lower connectivity changes than iTBS alone (Fig. 2E and Fig. 3B). Priming iTBS with cTBS did not result in any significant increases in connectivity. When comparing the two priming conditions directly to each other, we found significantly lower connectivity changes following cTBS–iTBS than iTBS–iTBS in both the occipital and temporal clusters (Fig. 2F and Fig. 3B, 3C).

**Table 1.** *F*-test results for the whole brain left DLPFC resting-state connectivity analysis.

		Cluster	Peak	MNI			
	*k*_E_	*p*_FWE-corr_	*F*	x	y	z	Region
1	179	0.000	8.85	-42	54	6	Frontal_Mid_2_L
			7.47	-38	44	4	Frontal_Inf_Tri_L
			7.46	-30	54	0	Frontal_Sup_2_L
2	72	0.042	8.65	58	-44	-4	Temporal_Mid_R
			6.16	60	-52	2	Temporal_Mid_R
3	82	0.019	7.57	-18	-88	0	Lingual_L
			5.52	-14	-98	-2	Calcarine_L
			5.18	-28	-94	-2	Occipital_Mid_L

Results of the F-test for the main effect of stimulation condition. For each statistically significant cluster, the cluster size (*k*_E_), FWE-corrected *p*-value (*p*_FWE-corr_), peak *F*-value, MNI peak coordinates, and corresponding anatomical region (Rolls et al., 2020) are provided. All clusters are thresholded with cluster-level family-wise error correction *p* < 0.05 with uncorrected voxelwise *p* < 0.005.

Abbreviations: MNI, Montreal Neurological Institute (MNI).

**Table 2.** Post hoc *t*-test comparisons.

		Cluster		Peak	MNI			
		*k*_E_	*p*_FWE-corr_	*T*	x	y	z	Region
iTBS alone > control								
	No significant clusters emerged							
iTBS alone < control								
	1	64	1.000	3.80	-18	-88	0	Occipital_Mid_L
				3.66	-16	-96	0	Occipital_Mid_L
iTBS-iTBS > iTBS alone								
	1	60	1.000	3.60	-18	-94	-2	Occipital_Mid_L
				3.58	-14	-100	2	Occipital_Mid_L
iTBS-iTBS < iTBS alone								
	1	89	0.994	4.14	-32	48	0	Frontal_Sup_2_L
				3.91	-34	42	6	Frontal_Inf_Tri_L
				3.56	-40	54	4	Frontal_Mid_2_L
cTBS-iTBS > iTBS alone								
	No significant clusters emerged							
cTBS-iTBS < iTBS alone								
	1	112	0.932	4.34	-30	40	8	Frontal_Mid_2_L
				3.89	-38	44	4	Frontal_Inf_Tri_L
				3.89	-28	52	0	Frontal_Sup_2_L
	2	67	0.997	4.10	62	-46	0	Temporal_Mid_R
				3.67	54	-50	4	Temporal_Mid_R
				3.16	54	-44	-4	Temporal_Mid_R
cTBS-iTBS > iTBS-iTBS								
	No significant clusters emerged							
cTBS-iTBS < iTBS-iTBS								
	1	70	0.990	4.18	56	-44	-2	Temporal_Mid_R
				3.68	60	-52	2	Temporal_Mid_R
	2	33	1.000	3.75	-20	-90	-2	Occipital_Mid_L
				3.25	-28	-94	-2	Occipital_Mid_L

Post hoc comparisons following the *F*-test results for the whole-brain left DLPFC resting-state connectivity analysis were conducted to specifically assess the effects of stimulation priming. Post hoc statistical parametric maps were masked using the *F*-test results, and significance was assessed at *p* < 0.00625 (i.e., 0.05/8). For each statistically significant cluster, the cluster size (*k*_E_), FWE-corrected *p*-value (*p*_FWE-corr_), peak *T*-value, MNI peak coordinates, and corresponding anatomical region (Rolls et al., 2020) are provided.

Abbreviations: cTBS, continuous theta burst stimulation; iTBS, intermittent theta burst stimulation; MNI, Montreal Neurological Institute (MNI).

### 3.3 Brain-behavior relationship

**Fig. 4.**
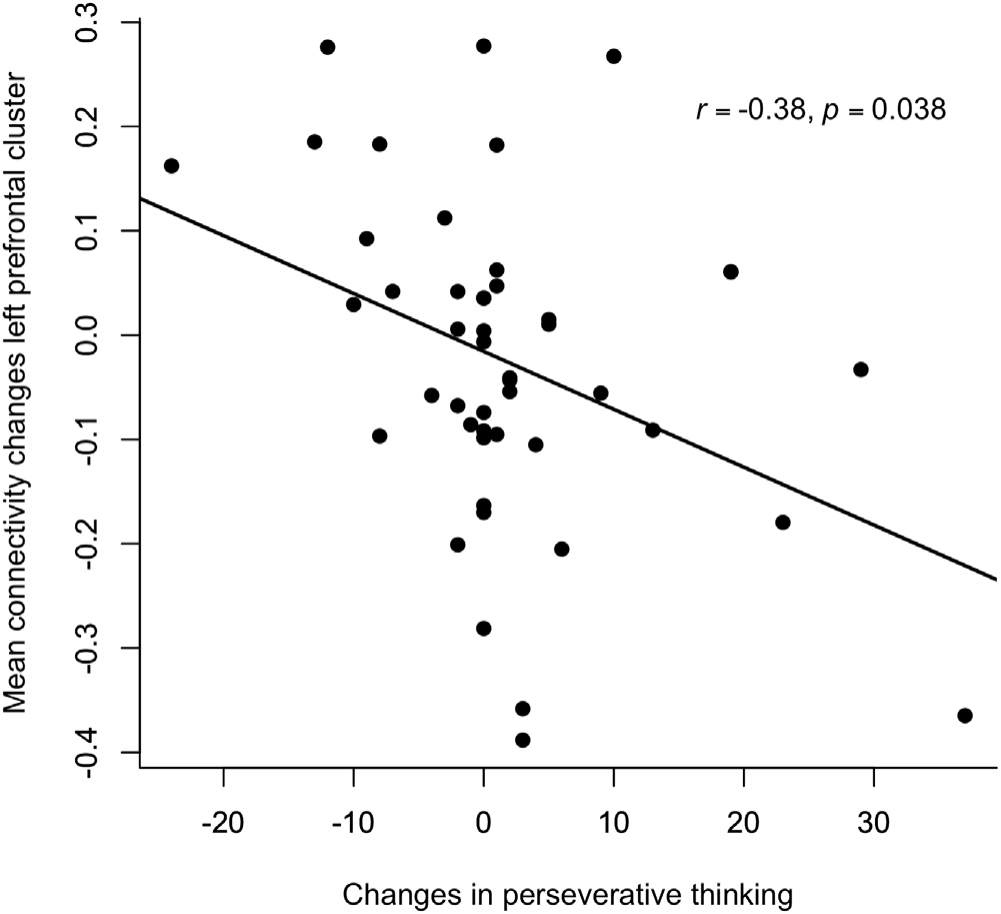
Association between changes in perseverative thinking and left prefrontal connectivity following cTBS-iTBS. Scatterplot demonstrating the negative association between changes in perseverative thinking and mean connectivity changes in the left prefrontal cluster following the cTBS-iTBS protocol Each dot represents an individual participant, and the solid line represents the linear fit. Both variables were calculated as post-stimulation minus baseline values, such that higher values reflect greater increases following stimulation. Greater increases in perseverative thinking were associated with stronger decreases in left prefrontal connectivity.

For the middle temporal and left occipital clusters, the linear mixed-effects models showed no significant effects of changes in mood or perseverative thinking on connectivity changes across stimulation conditions, all *X*^2^’s < 6.10, *p*’s < 0.110, *φ*’s > 0.36. For the frontal cluster, no effects of changes in mood were found, *X*^2^’s < 1.60, *p*’s > 0.660, *φ*’s < 0.18. However, a significant interaction between stimulation condition and changes in perseverative thinking was observed, *X*^2^ (3, N = 49) = 12.21, *p* = 0.040, *φ* = 0.51. Follow-up analyses indicated that this effect was driven by the cTBS-iTBS condition, *F*(1, 45) = 7.37, *p* = 0.038, *η_p_*^2^ = 0.14, such that higher increases in perseverative thinking were associated with stronger reductions in prefrontal connectivity (see Fig. 4). No signficant effects of changes in perseverative thinking were observed for the other stimulation conditions, all *F*’s < 2.41, *p*’s > 0.255, *η_p_*^2^’s < 0.05.

## 4 Discussion

In this study, we aimed to investigate how stimulation priming over the left DLPFC influences resting-state functional connectivity in healthy individuals. We applied two priming protocols: one using the same stimulation type for both prime and test (i.e., iTBS-iTBS), and one using different stimulation types (i.e., cTBS-iTBS). These were compared to an iTBS alone condition, as well as to each other. The main aim was to investigate significant changes in connectivity across these different priming conditions, in both local and distant brain regions and networks, and to identify which prime-test configuration would lead to the strongest increases in connectivity.

Contrary to earlier research findings, the standalone iTBS condition did not elicit significant changes in DLPFC-frontal or DLPFC-ACC connectivity (e.g., Chang et al., 2024; (Iwabuchi et al., 2017; Singh et al., 2020; Tang et al., 2019) when compared to the control condition (i.e., sham prime and test). Instead, we observed a significant reduction in connectivity changes in the left middle occipital gyrus. Notably, this is the same region where iTBS-iTBS priming led to increased connectivity changes relative to iTBS alone. However, mean connectivity changes in the iTBS-iTBS condition were comparable to the control condition, suggesting that the decrease observed with iTBS alone was attenuated or normalized by active priming, rather than fully reversed. This finding aligns with previous evidence that using the same stimulation protocol in a prime-test configuration (e.g., iTBS-iTBS) can weaken or even reverse after-effects (Mastroeni et al., 2013). Such outcomes are thought to reflect a metaplastic shift in the plasticity threshold, whereby the initial application of iTBS alters the excitability of the targeted network and modulates its subsequent responsiveness. Rather than enhancing the effects of iTBS, applying the same stimulation type as a prime (i.e., iTBS) may have dampened the impact of the test stimulation, consistent with metaplastic regulation. Further in line with these findings, we observed that iTBS-iTBS priming led to significantly lower connectivity changes in regions that are spatially close and highly interconnected with the stimulation site (i.e., the left DLPFC). Notably, our results demonstrate that these connectivity changes predominantly overlap with the seed region, likely reflecting direct modulation of local networks and highlighting an impact on short-range connections. These regions are involved in higher-order cognitive and emotional processing, with the superior, middle, and inferior frontal gyri playing key roles in executive functions (Friedman & Robbins, 2022). From a metaplasticity perspective, the decreased connectivity observed in these local circuits may reflect a form of activity-dependent regulation, whereby repeated excitatory input induces an inhibitory response. Notably, the reported effects were not present in the comparison between iTBS alone and the control condition, indicating that the observed changes do not simply reflect an additive effect of repeated stimulation. Instead, they likely represent non-linear, interaction-dependent outcomes specific to the priming configuration. This suggests that priming does not merely modulate the strength of a standalone iTBS session’s effect, but can instead alter the direction of the resulting connectivity changes. Rather than facilitating neuroplasticity in a way that increases functional connectivity, priming iTBS with iTBS may have shifted the system into a state that actively decreases connectivity, particularly within circuits involved in cognitive and emotional regulation.

With regard to cTBS-iTBS priming, our findings show that this type of priming did not result in significantly greater increases in connectivity compared to iTBS alone. Instead, it led to significantly reduced functional connectivity in both the stimulated (i.e., prefrontal) area as more distal, temporal regions. This outcome contradicts our initial hypothesis that combining different stimulation types (i.e., cTBS-iTBS) would lead to stronger increases in functional connectivity (versus iTBS alone) than using the same stimulation type for both prime and test (i.e., iTBS-iTBS). Notably, these findings parallel those observed with iTBS-iTBS priming, where reduced connectivity was also found in frontal regions suggesting that priming - whether with the same or a different type of stimulation - may not necessarily enhance connectivity in these areas, but could instead reduce functional connectivity within prefrontal networks. Importantly, these outcomes challenge classical metaplasticity frameworks, which posit that combining stimulation protocols expected to induce different plasticity-like effects, such as cTBS-iTBS, should shift the network into a more plastic, receptive state. According to this view, such a shift would ehance the response to the subsequent (test) iTBS protocol, typically reflected in increased functional connectivity. Instead, our results point to a more complex modulation, in which both cTBS-iTBS and iTBS-iTBS priming may decrease rather than increase functional connectivity. Moreover, priming iTBS with cTBS influenced connectivity in the middle temporal gyrus, a region that did not show any significant changes following either iTBS alone or iTBS-iTBS priming. Although mean connectivity changes in the cTBS-iTBS condition was slightly lower than in the control condition, this difference was not statistically significant. However, the significant difference between cTBS-iTBS and iTBS alone suggests that the cTBS prime altered how the network responded to subsequent iTBS. cTBS may have modulated the network’s plasticity threshold, shaping its response to subsequent iTBS in a way that led to reduced connectivity relative to the iTBS-alone condition. This may suggest that this priming combination alters connectivity dynamics beyond the prefrontal cortex, potentially modulating interactions between the frontoparietal control network and temporal regions involved in language and memory processing (Bohsali et al., 2025; Ferris et al., 2024). Given that the middle temporal gyrus has also been implicated in affective and social cognition (Pan et al., 2025; Wang et al., 2024), its reduced connectivity following cTBS-iTBS priming may further indicate a broader impact on networks supporting emotional and cognitive regulation. Overall, these findings suggests that prefrontal priming iTBS with cTBS may not facilitate subsequent iTBS-induced plasticity in the same way as observed in the motor cortex (Hassanzahraee et al., 2018), nor in the same manner as observed with prefrontal high- and low-frequency rTMS priming in depressed patients (Fitzgerald et al., 2008). Moreover, when comparing the two priming conditions directly to each other, we found significantly lower connectivity changes in the cTBS-iTBS condition relative to iTBS-iTBS in both the occipital and temporal clusters. This finding suggests that different priming configurations can differentially shape subsequent connectivity responses, with cTBS-iTBS priming leading to more pronouned connectivity reductions in these regions. This further supports the idea that priming may not universally enhance neuroplasticity but instead modulates connectivity in a complex, region- and state-dependent manner.

Furthermore, exploratory brain-behavior analyses revealed that changes in self-reported perseverative thinking, but not mood, significantly moderated stimulation-induced connectivity changes in the left prefrontal cluster, specifically following the cTBS-iTBS condition. Notably, these effects were observed despite the absence of group-level effects of stimulation condition on self-reported measures. This is consistent with our prior work on single-session TBS over the left DLPFC, where we also observed no group-level behavioural effects, while higher trait levels of rumination (i.e., a form of perseverative thinking) were associated with stronger cTBS-induced physiological responses (De Smet, Int-Veen, et al., 2024). Moreover, perseverative thinking has been linked to resting-state functional connectivity patterns showing engagement of medial prefrontal and cingulate regions (Makovac et al., 2020). Together, these findings suggest that variability in perseverative thinking may particularly influence prefrontal network responsiveness to stimulation. Notably, this effect was restricted to the cTBS-iTBS condition and was not observed for iTBS alone or iTBS-iTBS priming. While the mechanism underlying this condition-specific effect remains unclear, it may reflect differential sensitivity of stimulation protocols to ongoing psychological states. More broadly, these findings support emerging perspectives on brain-state dependency, suggesting that stimulation effects are shaped not only by prior stimulation (i.e., priming) but also by concurrent psychological states (De Smet, Razza et al., 2024; Schutter et al., 2023). From a methodological perspective, assessing psychological state via self-reports offers a practical approach to capturing relevant state fluctuations and may enhance the interpretability of stimulation effects (see also Di Rosa et al., 2024; Schutter et al., 2023). Future studies may benefit from systematically incorporating such measures to better understand and potentially reduce variability in neuromodulation responses.

Although speculative, one possible explanation for the overall observed reductions in connectivity following TBS priming might be that altering the stimulation history may have disrupted the homeostatic regulation of frontotemporal circuits, possibly leading to diminished functional coupling. Specifically, priming may interfere with the brain’s natural mechanisms for maintaining balanced connectivity within these networks. Homeostatic plasticity mechanisms ensure that connectivity remains within an optimal range, and external perturbations (such as priming) might trigger compensatory downregulation instead of facilitation (Karabanov et al., 2015; Miniussi et al., 2013). In patients, where connectivity is often considered dysregulated (Tura & Goya-Maldonado, 2023), TBS priming may induce a necessary shift toward normalization rather than causing disruption (Fox, Halko, et al., 2012). Furthermore, the present study examined the acute effects of a single session of TBS priming on functional connectivity, while clinical rTMS protocols typically involve repeated sessions over several weeks. Whereas single-session TBS priming may reduce connectivity, cumulative stimulation over time could lead to more lasting network reorganization and functional stability. However, using concurrent rTMS-fMRI, Tik et al. (2017) demonstrated that prefrontal 10 Hz rTMS-induced connectivity changes in healthy subjects can reflect meaningful neural adaptations relevant to therapeutic rTMS response. Nevertheless, while the mechanisms observed using a single session of TBS priming in healthy subjects are informative, they may not directly translate to outcomes in clinical populations (see also Chang et al., 2024).

An alternative interpretation of the present findings is that they reflect network- and context-dependent properties of TBS priming effects that are not fully captured by classical metaplasticity frameworks. These frameworks are largely informed by work on the motor cortex and typically assume that iTBS and cTBS exert intrinsically facilitatory and inhibitory effects on cortical plasticity, respectively (Hassanzahraee et al., 2018). However, accumulating evidence cautions against assuming that such principles generalize to prefrontal targets (Hermiller, 2025; Hussain & Freedberg, 2025). Meta-analytic evidence suggests that TBS effects in frontal regions are considerably more heterogenous, with both iTBS and cTBS producing excitatory and inhibitory effects on neural measures across resting-state and task-based contexts (Kirkovski et al., 2023). In particular, while motor cortex stimulation often shows relatively consistent patterns of connectivity modulation at rest, frontal stimulation appears far more sensitive to contextual factors such as cognitive state and the network engaged (Kirkovski et al., 2023). This variability has been attributed, at least in part, to the complex neural architecture and functional organization of the prefrontal cortex, which is embedded in multiple overlapping large-scale networks, supports diverse cognitive, affective, and social processes, and exhibits highly flexible patterns of engagement depending on context (Kolb et al., 2012; O’Reilly, 2010). Consistent with this view, recent fMRI connectivity work demonstrates that TBS effects depend on stimulation rhythm, targeted network, and cognitive context, and are not adequately captured by simple excitatory-inhibitory models (Hermiller, 2024). Together, these observations argue for a shift away from polarity-based models toward more nuanced frameworks that explicitly account for network architecture and contextual influences when interpreting TBS effects.

While this study has several strengths, including its rigorous design and relatively large sample size, several limitations should be considered. First, the timing between priming and test stimulation appears to be a crucial factor in determining the direction and magnitude of aftereffects (Tse et al., 2018). Based on evidence from motor cortex studies (Wittkopf et al., 2021) and the timing used in clinical protocols (De Witte et al., 2025; Duprat et al., 2016), we implemented a 15-minute interval between priming and test TBS. This timing window is thought to be fundamental for inducing homeostatic responses, however, the optimal intersession interval remains uncertain (Thomson & Sack, 2020). In fact, some studies on motor cortex plasticity have reported conflicting effects when two iTBS sessions are repeated with short intervals (i.e., less than 30 minutes, Wittkopf et al., 2021), with some evidence suggesting that additive LTP-like plasticity may be promoted, while others indicate homeostatic metaplasticity mechanisms may instead stabilize neural excitability (Thomson & Sack, 2020; Wittkopf et al., 2021). In the prefrontal cortex, applying two consecutive iTBS sessions with a 5-minute interval has been associated with inhibitory rather than facilitatory effects on stress regulation (De Witte et al., 2022). This raises the question of whether the 15-minute interval used in our study was optimal or whether a different, longer intersession timing (such as 50 to 60 minutes, see Thomson & Sack, 2020) might have yielded different effects. Second, in addition to the timing between priming and test stimulation, the interval between stimulation and neuroimaging assessment may also influence the observed connectivity effects. In the present study, resting-state fMRI was acquired approximately 10 minutes after stimulation, and we cannot determine whether connectivity changes would have differed at earlier or later time points. Previous work suggests that iTBS-induced functional connectivity changes can be transient and evolve over time. For example, Tang et al. (2019) reported immediate post-iTBS increases in connectivity between frontal regions (e.g., the left dorsolateral superior frontal gyrus) alongside decreases in orbital gyrus connectivity, with these effects weakening after approximately 15 minutes. Similarly, Singh et al. (2020) observed an initial increase in connectivity within the rostral ACC at 10-15 minutes post-stimulation, followed by a decrease in connectivity between rostral and dorsal ACC at later time points. As such, the effects observed here should be interpreted within the specific post-stimulation window in which these connectivity changes were assessed. Future studies incorporating multiple post-stimulation imaging time points, or employing online TMS-fMRI approaches, may help to disentangle immediate from delayed effects of TBS and provide a more comprehensive understanding of how priming influences functional connectivity over time. Third, we lack insight into the neurophysiological processes occurring during the 15-minute rest interval. A concurrent TMS-fMRI setup could have provided more insight into how TBS priming influences network activity, not only over time but also during the rest period between priming and test stimulation. Keeping participants in the scanner throughout the priming protocol would also have allowed for the examination of neural dynamics unfolding during this critical interval between prime and test. Although implementing an entire iTBS protocol under continuous MRI acquisition remains technically challenging, recent advancements (Chang et al., 2024) offer promising avenues for future studies to better capture how TBS priming dynamically shapes large-scale network activity over time.

To conclude, these results highlight the complexity of prefrontal TBS priming effects on resting-state functional connectivity. Contrary to our expectations, neither priming iTBS with the same stimulation protocol (iTBS-iTBS) nor with the opposite protocol (cTBS-iTBS) led to increases in connectivity. Instead, iTBS-iTBS priming was associated with reduced connectivity changes in prefrontal regions, while cTBS-iTBS led to reductions in both prefrontal and temporal areas. Moreover, cTBS-iTBS priming resulted in significantly greater reductions in connectivity than iTBS-iTBS in both temporal and occipital regions, suggesting a more pronounced and spatially extended decrease in connectivity than that observed with iTBS-iTBS priming. These priming effects were further modulated by psychological state, as changes in perseverative thinking were associated with variability in prefrontal connectivity responses following cTBS-iTBS priming. Together, these findings indicate complex interactions between stimulation history (i.e., priming) and concurrent psychological states in shaping connectivity outcomes. Future research should further explore the mechanisms driving these effects.

## Supplementary Material

Supplementary Material

## Data Availability

Due to the requirements of the institutional ethics, the datasets generated and analyzed in the current study are only made available via a request to the corresponding author, with the need a formal data-sharing agreement.
