# Supplementary material for: Stimulation priming and psychological state shape functional connectivity following prefrontal theta-burst stimulation
Source: Imaging Neurosci (Camb). 2026 Jun 4;4:IMAG.a.1267. doi: 10.1162/IMAG.a.1267 (PMC13237992; doi:10.1162/IMAG.a.1267)
Supplement: Supplementary Material [file IMAG.a.1267_supp.pdf]

Stimulation priming and psychological state shape functional connectivity following prefrontal theta-burst stimulation

Stefanie De Smet, Guo-Rong Wu, Debby C.W. Klooster, Beatriz Catoira, Sara De Witte, Lais B. Razza, Vincent van de Ven, Alexander T. Sack, Chris Baeken, Marie-Anne Vanderhasselt

Correspondence

Stefanie De Smet, M.Sc., Department of Head and Skin Psychiatry and Medical Psychology, Ghent University Hospital, Corneel Heymanslaan 10 – 9000 Ghent, Belgium; E-mail: stefaniedesmet.phd@gmail.com

Supplementary material

2. Material and method

2.1. Study sample

Participants met the following inclusion criteria: (1) aged between 18 and 35 years, (2) right-handed, (3) no history of or current psychiatric or neurological conditions, (4) no cardiovascular diseases, (5) no use of substances or medications influencing mood, cognition, or cardiovascular function (such as antidepressants, benzodiazepines, or Z-drugs), (6) smoking fewer than 10 cigarettes per day, (7) normal or corrected-to-normal vision, (8) no history of severe head trauma, brain surgery, or epileptic seizures, (9) no metal implants or fragments in the head, (10) no cochlear implants or pacemakers, (11) not pregnant, (12) no contraindications for MRI or TMS, and (13) no prior exposure to TMS.

2.3. Adverse effects

Adverse effects were systematically assessed using 9 self-report questions assessing transient hyperactivity, impulsivity, or irritability; headache; local pain; neck pain; tooth pain; tingling sensations; hearing changes; transient cognitive or neuropsychological changes (memory and attention); and irritation at the stimulation site (responses ranging from “0 = not at all” to “4 = very much”).

Table 1. Adverse effects.

	**Sham prime + sham test**	**Sham prime + iTBS test**	**iTBS prime + iTBS test**	**cTBS prime + iTBS test**	**Statistics**
**Transient feelings of hyperactivity or impulsivity**	0.44 (0.79)	0.52 (0.84)	0.58 (0.67)	0.62 (0.90)	F(3, 196) = 0.47, p = 0.788, $\eta^2 < 0.01$
**Headache**	0.52 (0.89)	0.64 (1.01)	0.88 (1.19)	0.76 (0.94)	F(3, 196) = 1.17, p = 0.668, $\eta^2 = 0.02$
**Local pain**	0.52 (0.97)	0.78 (1.13)	1.10 (1.28)	1.06 (1.25)	F(3, 196) = 1.69, p = 0.251, $\eta^2 = 0.04$

**Neck pain**	0.44 (0.73)	0.42 (0.64)	0.40 (0.70)	0.60 (0.86)	$F(3, 196) = 0.77$, $p = 0.668$, $\eta^2 = 0.01$
**Tooth pain**	0.12 (0.39)	0.12 (0.44)	0.34 (0.85)	0.20 (0.64)	$F(3, 196) = 1.47$, $p = 0.667$, $\eta^2 = 0.02$
**Tingling sensations**	0.86 (1.13)	0.84 (0.96)	1.14 (1.11)	0.82 (1.00)	$F(3, 196) = 1.03$, $p = 0.668$, $\eta^2 = 0.02$
**Hearing changes**	0.20 (0.61)	0.12 (0.52)	0.22 (0.55)	0.20 (0.57)	$F(3, 196) = 0.31$, $p = 0.817$, $\eta^2 < 0.01$
**Transient cognitive & neuropsychological changes**	0.32 (0.65)	0.24 (0.52)	0.44 (0.76)	0.36 (0.75)	$F(3, 196) = 0.76$, $p = 0.666$, $\eta^2 = 0.01$
**Irritation at the stimulation site**	0.46 (1.01)	0.78 (1.09)	1.10 (1.31)	0.84 (1.20)	$F(3, 196) = 2.57$, $p = 0.251$, $\eta^2 = 0.04$

Mean (SD, i.e. standard deviation) for the self-report items assessing adverse effects to the four stimulation protocols. Overall, there were no significant differences. Abbrev.: cTBS, continuous theta burst stimulation, cTBS; iTBS, intermittent theta burst stimulation.

2.5. Data plan

2.5.1. Imaging data analyses

Functional data were realigned using SPM realign & unwarp procedure (Andersson et al., 2001) integrating fieldmaps for susceptibility distortion correction, where all scans were coregistered to a reference image (first scan of the first session) using a least squares approach and a 6 parameter (rigid body) transformation, and resampled using b-spline interpolation (Friston et al., 1995) to simultaneously correct for motion, magnetic susceptibility geometric distortions, and their interaction. Temporal misalignment between different slices of the functional data (acquired in interleaved Siemens order) was corrected following SPM slice-timing correction (STC) procedure (Sladky et al., 2011), using sinc temporal interpolation to resample each slice BOLD time series to a common mid-acquisition time. Potential outlier scans were identified using ART (Whitfield-Gabrieli et al., 2011) as acquisitions with framewise displacement above 0.9 mm or global BOLD signal changes above 5 standard deviations (Nieto-Castanon, 2022; Power et al., 2014). Functional and anatomical data were coregistered and normalized into standard MNI space, segmented into grey matter, white matter, and CSF tissue classes, and resampled to 2 mm isotropic voxels following an indirect normalization procedure (Calhoun et al., 2017) using SPM unified segmentation and normalization algorithm (Ashburner, 2007; Ashburner & Friston, 2005) with the default IXI-549 tissue probability map template. Last, functional data were smoothed using spatial convolution with a Gaussian kernel of 4 mm full width half maximum (FWHM).

In addition, functional data were denoised using a standard denoising pipeline (Nieto-Castanon, 2020) including the regression of potential confounding effects characterized by white matter timeseries (5 CompCor noise components), CSF timeseries (5 CompCor noise components), motion parameters and their first order derivatives (12 factors) (Friston et al., 1996), outlier scans (below 70 factors)(Power et al., 2014), and linear trends (2 factors) within each functional run, followed by bandpass frequency filtering of the BOLD time series

(Hallquist et al., 2013) between 0.008 Hz and 0.09 Hz. CompCor (Behzadi et al., 2007; Chai et al., 2012) noise components within white matter and CSF were estimated by computing the average BOLD signal as well as the largest principal components orthogonal to the BOLD average, motion parameters, and outlier scans within each subject's eroded segmentation masks. From the number of noise terms included in this denoising strategy, the effective degrees of freedom of the BOLD signal after denoising were estimated to range from 0 to 622.8 (average 443.7) across all subjects (Nieto-Castanon, 2022). Additional checks were conducted to identify participants with excessive motion, based on the percentage of outlier scans detected using ART (Power et al., 2014). Participants with more than 10% outlier scans (n = 3) were excluded from the analysis to maintain high data quality.

Functional connectivity strength was represented by Fisher-transformed bivariate correlation coefficients from a weighted general linear model (Nieto-Castanon, 2020), estimated separately for each seed area and target voxel, modeling the association between their BOLD signal time series. Individual scans were weighted by a boxcar signal characterizing each experimental condition convolved with an SPM canonical hemodynamic response function and rectified. The SBC maps were used to compute delta scores by subtracting the pre-stimulation maps from the post-stimulation SBC maps for each of the four stimulation conditions. This was done in R (version 4.4.0, 2024-04-24, "Puppy Cup") within the RStudio environment (version 2024.12.0+467), using the ‘Neurobase’ package (Muschelli, 2024).

3. Results

3.1. Self-report measures

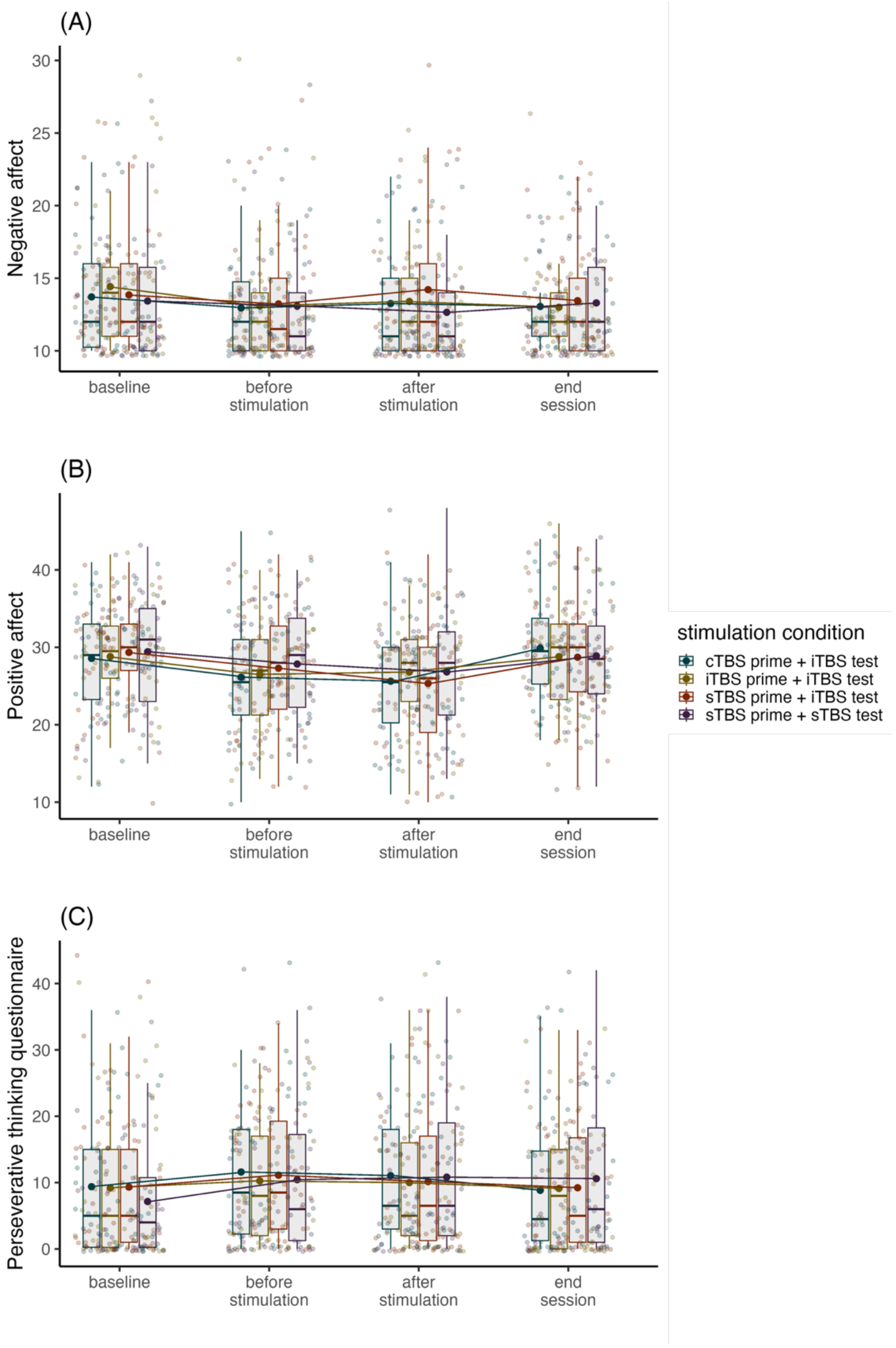

Figure 1. Raincloud plots for (A) negative affect, (B) positive affect and (C) perseverative thinking. Individual jittered raw data is represented by dots. Each boxplot displays the stimulation condition median alongside the interquartile ranges (horizontal lines). Bold dots in the boxplots represent the estimated marginal mean of the fitted models.

4. References

Andersson, J. L. R., Hutton, C., Ashburner, J., Turner, R., & Friston, K. (2001). Modeling Geometric Deformations in EPI Time Series. *NeuroImage*, *13*(5), 903–919. https://doi.org/10.1006/nimg.2001.0746

Ashburner, J. (2007). A fast diffeomorphic image registration algorithm. *NeuroImage*, *38*(1), 95–113. https://doi.org/10.1016/j.neuroimage.2007.07.007

Ashburner, J., & Friston, K. J. (2005). Unified segmentation. *NeuroImage*, *26*(3), 839–851. https://doi.org/10.1016/j.neuroimage.2005.02.018

Behzadi, Y., Restom, K., Liau, J., & Liu, T. T. (2007). A component based noise correction method (CompCor) for BOLD and perfusion based fMRI. *NeuroImage*, *37*(1), 90–101. https://doi.org/10.1016/j.neuroimage.2007.04.042

Calhoun, V. D., Wager, T. D., Krishnan, A., Rosch, K. S., Seymour, K. E., Nebel, M. B., Mostofsky, S. H., Nyalakanai, P., & Kiehl, K. (2017). The impact of T1 versus EPI spatial normalization templates for fMRI data analyses. *Human Brain Mapping*, *38*(11), 5331–5342. https://doi.org/10.1002/hbm.23737

Chai, X. J., Castañón, A. N., Öngür, D., & Whitfield-Gabrieli, S. (2012). Anticorrelations in resting state networks without global signal regression. *NeuroImage*, *59*(2), 1420–1428. https://doi.org/10.1016/j.neuroimage.2011.08.048

Ehring, T., Raes, F., Weidacker, K., & Emmelkamp, P. M. G. (2012). Validation of the Dutch Version of the Perseverative Thinking Questionnaire (PTQ-NL). *European Journal of Psychological Assessment*, *28*(2), 102–108. https://doi.org/10.1027/1015-5759/a000097

Ehring, T., Zetsche, U., Weidacker, K., Wahl, K., Schönfeld, S., & Ehlers, A. (2011). The Perseverative Thinking Questionnaire (PTQ): Validation of a content-independent measure of repetitive negative thinking. *Journal of Behavior Therapy and Experimental Psychiatry*, *42*(2), 225–232. https://doi.org/10.1016/j.jbtep.2010.12.003

Friston, K. J., Williams, S., Howard, R., Frackowiak, R. S. J., & Turner, R. (1996).

Movement-Related effects in fMRI time-series. *Magnetic Resonance in Medicine*, *35*(3), 346–355. https://doi.org/10.1002/mrm.1910350312

Friston, Karl. J., Ashburner, J., Frith, C. D., Poline, J. -B., Heather, J. D., & Frackowiak, R. S. J. (1995). Spatial registration and normalization of images. *Human Brain Mapping*, *3*(3), 165–189. https://doi.org/10.1002/hbm.460030303

Hallquist, M. N., Hwang, K., & Luna, B. (2013). The nuisance of nuisance regression: Spectral misspecification in a common approach to resting-state fMRI preprocessing reintroduces noise and obscures functional connectivity. *NeuroImage*, *82*, 208–225. https://doi.org/10.1016/j.neuroimage.2013.05.116

Muschelli, J., 2024. neurobase: 'Neuroconductor' Base Package with Helper Functions for 'nifti' Objects. R package version 1.32.4, https://CRAN.R-project.org/package=neurobase

Nieto-Castanon, A. (2020). *Handbook of functional connectivity Magnetic Resonance Imaging methods in CONN*. Hilbert Press. https://doi.org/10.56441/hilbertpress.2207.6598

Nieto-Castanon, A. (2022). *Preparing fMRI Data for Statistical Analysis* (Version 1). arXiv. https://doi.org/10.48550/ARXIV.2210.13564

Power, J. D., Mitra, A., Laumann, T. O., Snyder, A. Z., Schlaggar, B. L., & Petersen, S. E. (2014). Methods to detect, characterize, and remove motion artifact in resting state fMRI. *NeuroImage*, *84*, 320–341. https://doi.org/10.1016/j.neuroimage.2013.08.048

Sladky, R., Friston, K. J., Tröstl, J., Cunnington, R., Moser, E., & Windischberger, C. (2011). Slice-timing effects and their correction in functional MRI. *NeuroImage*, *58*(2), 588–594. https://doi.org/10.1016/j.neuroimage.2011.06.078

Watson, D., Clark, L. A., & Carey, G. (1988). Positive and negative affectivity and their relation to anxiety and depressive disorders. *Journal of Abnormal Psychology*, *97*(3), 346–353. https://doi.org/10.1037/0021-843X.97.3.346

Whitfield-Gabrieli, S., Nieto-Castanon, A., & Ghosh, S. (2011). Artifact detection tools (ART). Cambridge, MA. Release Version, 7(19), 11.
